# Mechanism of phosphate removal from aqueous solutions by biochar supported nanoscale zero-valent iron†

**DOI:** 10.1039/d0ra07391a

**Published:** 2020-10-26

**Authors:** Fengfeng Ma, Baowei Zhao, Jingru Diao, Yufeng Jiang, Jian Zhang

**Affiliations:** School of Environmental and Municipal Engineering, Lanzhou Jiaotong University No. 88, West Anning Road Lanzhou 730070 Gansu China mayibo1985@126.com +86-15117004233

## Abstract

The purpose of this study was to investigate the removal mechanism of phosphate by rape straw biochar (RSBC) supported nanoscale zero-valent iron (nZVI). BET, TEM, FTIR and XPS characterizations of the composite material (nZVI-RSBC) indicated that nZVI was successfully supported on the RSBC, and nZVI-RSBC had a high specific surface area and abundant oxygen-containing functional groups. Batch experiments showed that the adsorption data could be fitted well with the Sips isotherm model and pseudo-second-order kinetic model, suggesting that phosphate adsorption onto RSBC and nZVI-RSBC was due to surface and chemical processes. The maximum adsorption capacities of RSBC and nZVI-RSBC for phosphate obtained by the Sips isotherm model fitting were 3.49 mg g^−1^ and 12.14 mg g^−1^, respectively. The pH value of the solution greatly affected the adsorption capacity of nZVI-RSBC for phosphate. The combined results of batch experiments and characterizations revealed that the possible mechanism was the complexation of oxygen-containing functional groups on the surface of nZVI-RSBC with phosphate, hydrogen bonding, and electrostatic attraction between phosphate and the positively charged adsorption sites under acidic conditions. Such a strong adsorption capacity, as well as the characteristics of easy availability, excellent recyclability and low cost, make nZVI-RSBC potentially suitable for the treatment of phosphate-rich water.

## Abstract 2

The purpose of this study was to investigate the removal mechanism of phosphate by rape straw biochar (RSBC) supported nanoscale zero-valent iron (nZVI).

## Graphical abstract

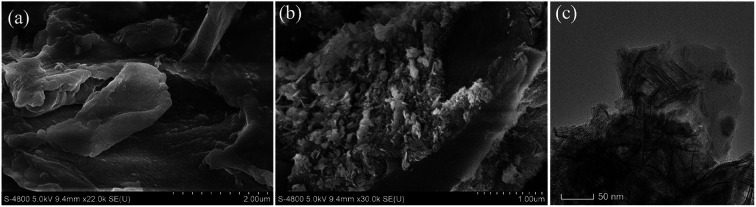

## 1. Introduction

The excessive discharge of phosphorus into surface and groundwater causes eutrophication.^1,2^ On the other hand, phosphorus is one of the primary nutrients required for the metabolism of biological species.^3^ Therefore, the recovery or removal of phosphate from wastewater has attracted more and more attention, and technology for the effective removal or recovery of phosphate from wastewater has been extensively studied.^4^ Conventional phosphate removal technologies mainly include biological removal^5^ and physico-chemical removal, such as membrane techniques, ion-exchange, coagulation and adsorption.^2^ However, chemical treatment not only creates the problem of solid waste disposal, but also has a relatively high cost and cannot be further processed to meet the demand for drinking water. Moreover, an additional sedimentation tank is required during the chemical treatment process.^6^ Although chemical treatment has a relatively high efficiency in removing phosphate, it may bring unexpected anions.^7^ Because environmental conditions (such as pH, temperature, dissolved oxygen content, and hydrodynamics) have a great influence on the growth and reproduction of microorganisms, biological treatment is challenging to control, which often leads to unstable phosphorus concentrations in wastewater.^8^ Among the phosphate removal technologies, adsorption technology has attracted widespread attention as a promising phosphate removal technology because of its simplicity, durability and cost-effectiveness.

nZVI has been widely used to remove antibiotics, dyes, heavy metals, and other pollutants in the environment due to its environmental friendliness, simple use and low cost.^9^ Many researches^1,10–12^ have reported that nZVI has a strong removal performance for phosphate. Although nZVI has the characteristics of a large specific surface area, high reactivity and cost efficiency, its reliability in environmental applications is still worrying.^13^ nZVI particles are fine powders, which can easily reunite with each other to form a necklace-like structure, preventing certain reactive sites from participating in the contaminant removal process.^14^ The use of nZVI in the wastewater remediation process may cause other environmental pollution problems, such as the rapid loss of nZVI and iron pollution in drinking water.^15^ To directly apply nZVI to water treatment processes (such as conventional treatment processes), nZVI needs to be quickly and completely separated by gravity settling for recover, reuse, and to improve the quality of wastewater.^16^ Therefore, nZVI should be carried on a support material to overcome the agglomeration of nZVI particles and enhance their separation and hydraulic conductivity.

Biochar is a carbon-rich particle obtain from the pyrolysis of various biomass in an oxygen-limited condition, and it is cheaper than activated carbon.^17^ Biochar has the characteristics of rich oxygen-containing functional groups and high specific surface area.^18^ Many studies have shown that biochar can be used as an excellent adsorbent for the removal of pollutants in the environment.^14,18–20^ However, the isoelectric point (IEP) of typical biochar is usually 2–3,^19^ and phosphate is negatively charged at most pH values of solution. Thus, the adsorption capacity of biochar towards phosphate is poor due to electrostatic repulsions.^21^ For instance, Jung *et al.*^22^ reported that the maximum adsorption capacity of biochar for phosphate was 6.79 mg g^−1^. Thus, it is necessary to modify biochar to change its surface charge properties and improve its adsorption effect on phosphate. To expand and improve the adsorption performance of nZVI and biochar, nZVI particles can be supported on the surface of biochar to change its IEP and enhance the affinity towards phosphate in an extensive pH range.^23^ Furthermore, the modification can also prevent the aggregation of nZVI particles and increase the reactivity of nZVI particles, hence increasing the exposure of their reaction sites and their adsorption capacity for phosphate. Although nZVI supported biochar has been widely used to remove organic pollutants and heavy metals from wastewater,^14,18,19^ to our best knowledge, few papers have discussed the removal of phosphate by nZVI supported biochar.

In this study, the composite material (nZVI-RSBC) of rape straw biochar (FSBC) supported nanoscale zero-valent iron (nZVI) was synthesized for phosphate removal, and the removal performance and mechanism of nZVI-RSBC towards phosphate were investigated. The specific objectives were (1) to characterize the physical and chemical properties of RSBC and nZVI-RSBC by SEM, TEM, TGA, BET, FTIR and XPS, (2) to evaluate the phosphate adsorption performance of nZVI-RSBC and determine the effect of initial pH value of the solution on the removal of phosphate, and (3) to elucidate the removal mechanism of phosphate by FTIR and XPS results. The results of this study will provide valuable information for the application of nZVI-based supporting materials in the treatment of phosphate-containing water.

## 2. Materials and methods

### 2.1. Preparation of RSBC and nZVI-RSBC

Rape straw was collected from Lanzhou, Gansu. The preparation method of rape straw biochar (RSBC) was similar to a method reported previously.^24^ nZVI-RSBC was prepared by a liquid-phase reduction method. Briefly, 12.44 g of FeSO_4_·7H_2_O was added to 250 mL of deionized water. Then, the RSBC was dispersed in the above solution with stirring for 1 hour. Next, 250 mL of NaBH_4_ solution (0.55 mol L^−1^) was slowly added dropwise under vigorous stirring. The entire preparation process was carried out under nitrogen protection. Then, the suspension was filtered using a vacuum pump, and the solid was washed with 300 mL of ethanol three times and vacuum dried. The obtained material was denoted nZVI-RSBC, and nZVI-RSBC was packed in a sealed glass bottle protected by nitrogen to prevent oxidation of the sample.

### 2.2. Characterization

The elemental contents were determined using an element analyzer (Vario EL cube, Heraeus, Germany). The surface morphologies were observed by scanning electron microscopy (JSM-5600 LV, Japan), and transmission electron microscopy (TEM) images were taken by a Talos F200S (Holland) to investigate the size and dispersion of nZVI on the RSBC. The Brunauer–Emmett–Teller specific surface area (BET SSA) and pore size distribution were determined by a surface characterization analyzer (Micromeritics ASAP 2010, USA) using N_2_ adsorption at 77 K. Thermal stability of RSBC and nZVI-RSBC were determined using thermogravimetric analysis (TGA, Mettler Toledo, Switzerland). The functional groups and chemical structures were identified by Fourier transform infrared spectroscopy (FTIR, Nexus870, USA). The surface composition and valence states of the elements were determined by X-ray photoelectron spectroscopy (ESCALAB 250 Xi, Thermo Fisher Scientific, USA).

### 2.3. Batch adsorption experiments

Batch experiments for phosphate were carried out under batch conditions: 0.05 g of nZVI-RSBC (or RSBC) was shaken with 20 mL of phosphate solution at pH 7.0, at a concentration between 5 and 60 mg L^−1^, at a controlled temperature of 25 °C. In adsorption kinetic experiments, 0.05 g of RSBC (or nZVI-RSBC) was added into 20 mL phosphate solution (pH = 7.0 ± 0.1, 20 mg L^−1^). Then the mixtures were agitated at different times (0.17–24 h) in a shaker (CHA-S Shaker, China) at 150 rpm and 25 °C. The mixed samples were filtered with a 0.45 μm filter membrane. The phosphate concentration was determined by the molybdenum blue colorimetric method.^25^ The adsorbing capacity (*q*, mg g^−1^) of phosphate was calculated by:1
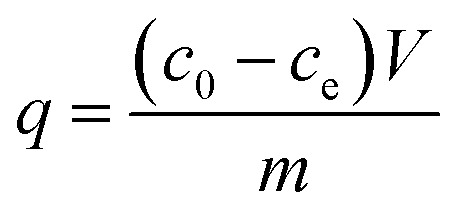
where *c*_0_ and *c*_e_ are the initial and equilibrium concentrations (mg L^−1^), respectively, *V* is the experimental solution volume (L), and *m* is the mass of adsorbent (g).

The influence of initial solution pH on phosphate removal by RSBC and nZVI-RSBC was examined in the range from 2.0 to 10.0, which was adjusted by 0.1 M NaOH and HCl. The initial concentration of phosphate was fixed at 50 mg L^−1^.

### 2.4. Recyclability tests

First, to evaluate the recyclability of RSBC and nZVI-RSBC, phosphate adsorption tests were carried out under the initial phosphate concentration of 50 mg L^−1^ (other conditions were the same as the batch adsorption experiments). Next, after reaching the equilibrium stage, the adsorbent was immediately collected from the solution by vacuum filtration. Then, the collected adsorbent was immersed in 1.0 M NaOH solution. The mixture was stirred at 150 rpm for 10 hours at 25 °C. 6 cycles of the recyclability test were repeated, and eqn (1) was used to determine the amount of phosphate adsorbed.

### 2.5. Adsorption models

The adsorption kinetics data were fitted with the pseudo-first-order model, pseudo-second-order model, Elovich model, and intra-particle diffusion model. The adsorption isotherm data were fitted with Langmuir, Freundlich, Sips, and Temkin models. All the adsorption models are listed in Table 1.

**Table:** List of adsorption kinetic and isotherm models used in this study^a^

Models	Expression
Pseudo-first-order	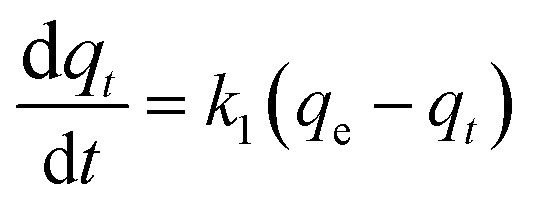
Pseudo-second-order	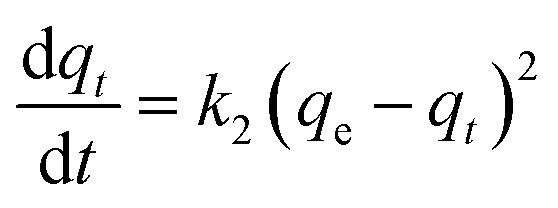
Elovich	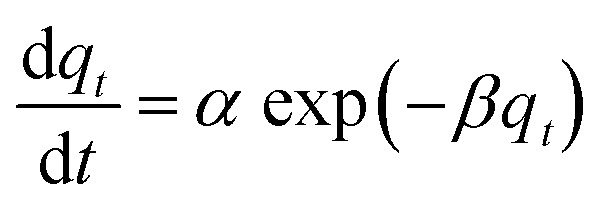
Intra-particle diffusion	*q*_*t*_ = *k*_d_*t*^1/2^ + *C*_i_
Langmuir	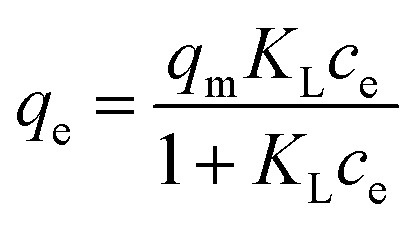
Freundlich	*q*_e_ = *K*_F_*c*_e_^1/*n*^
Sips	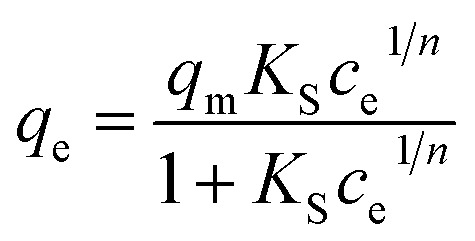
Temkin	*q*_e_ = *A *ln* K*_*t*_*c*_e_

a Where *q*_*t*_ and *q*_e_ (mg g^−1^) are the amounts of phosphate adsorbed at time *t* (h) and equilibrium, respectively; *k*_1_ (h^−1^), *k*_2_ (g mg^−1^ h^−1^), and *k*_d_ (mg g^−1^ h^−1/2^) are the pseudo-first-order, pseudo-second-order, and intra-particle diffusion rate constants, respectively; *α* (mg g^−1^ h^−1^) is the initial adsorption rate, *β* (g mg^−1^) is the desorption constant, and *C*_i_ is a constant related to the boundary layer thickness; *K*_L_ (L mg^−1^), *K*_F_ (mg g^−1^), and *K*_S_ (L mg^−1^) are the Langmuir, Freundlich, and Sips constants, respectively. 1/*n* is the constant indicative of the intensity of the adsorption. *A* (L mg^−1^) and *K*_*t*_ (J mol^−1^) are Temkin constants.

## 3. Results and discussion

### 3.1. Characterizations of RSBC and nZVI-RSBC

**Fig. 1:**
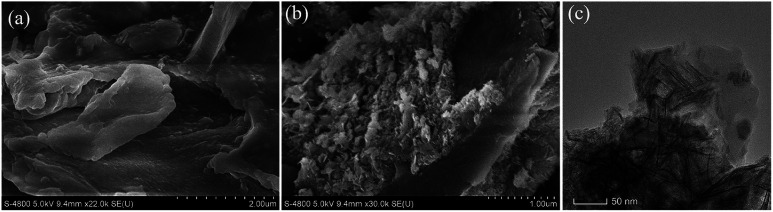
SEM images of RSBC (a) and nZVI-RSBC (b); TEM image of the nZVI-RSBC (c).

The SEM and TEM images of RSBC and nZVI-RSBC are presented in Fig. 1. It can be seen that the SEM image of RSBC is relatively smooth, but the pore structure is not abundant (Fig. 1a). As shown in Fig. 1b, nZVI particles were evenly distributed on the surface of RSBC. Moreover, the nZVI particles were equably dispersed on the surface of nZVI-RSBC and rarely agglomerated. It can be seen from the TEM image of nZVI-RSBC (Fig. 1c) that nZVI was dispersed on the surface of RSBC without agglomeration, and the nZVI particles were spherical with a relatively uniform size of 20–30 nm, which indicated that nZVI particles were well supported on the surface of RSBC. This phenomenon showed that RSBC could inhibit the agglomeration of nZVI and improve its dispersion and stability.^9^

**Fig. 2:**
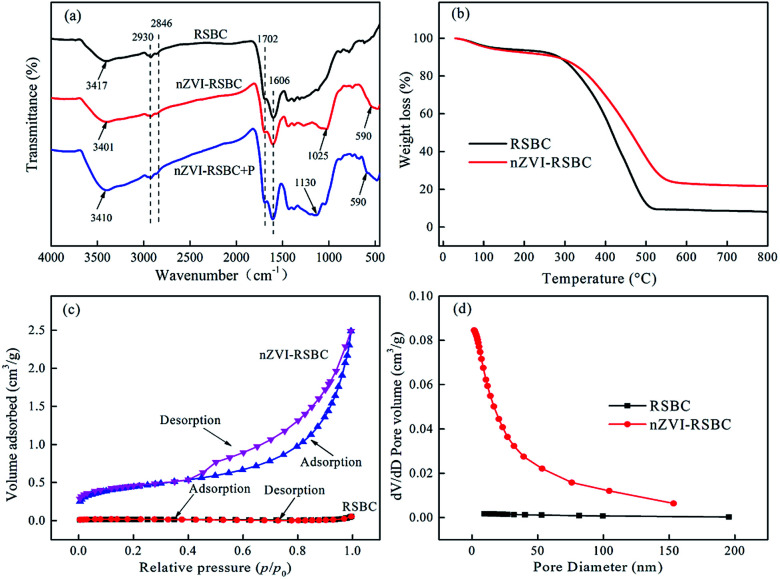
FTIR spectrum of RSBC, nZVI-RSBC, and nZVI-RSBC with phosphate adsorption (a); thermogravimetric (TG) curves of RSBC and nZVI-RSBC (b); N_2_ adsorption–desorption isotherms (c) and pore size distributions of RSBC and nZVI-RSBC (d).

FTIR spectra of RSBC, nZVI-RSBC, and nZVI-RSBC with phosphate are obtained in the range of 4000–400 cm^−1^ (Fig. 2a). The characteristic peaks at 2930 cm^−1^ and 2846 cm^−1^ correspond to the –CH_2_– groups. The bands at 1702 cm^−1^ and 1606 cm^−1^ can be assigned to the C=O and C=C groups, respectively.^26^ The band at 590 cm^−1^ could be ascribed to the Fe–O stretch vibration of Fe_2_O_3_ and Fe_3_O_4_, indicating that nZVI particles loaded on the RSBC were partially oxidized.^27^ After the supporting of nZVI onto the RSBC, the band at 3417 cm^−1^ shifted to a lower wavenumber of 3401 cm^−1^. This shift may be due to the coordination reaction between the lone pair of electrons on the O atom and the 3d space orbital on the Fe atom, which leads to the decrease of the electron cloud density and the bonding force constant of the O–H group, thereby weakening the O–H stretch vibration, and the Fe–O bond was strengthened.^20^ The characteristic peak at 3401 cm^−1^ before adsorption attributed to O–H shifted to 3410 cm^−1^ after the adsorption, and the transmittance was significantly enhanced, which may be due to the conversion of nZVI on the surface of nZVI-RSBC to FeOOH.^28^ The disappearance of the characteristic peak at 1025 cm^−1^ and a new characteristic peak at 1130 cm^−1^ for nZVI-RSBC after phosphate adsorption were attributed to the asymmetric vibration of Fe–O–P, indicating that chemical reaction occurred between Fe and phosphate.^8^ The above results indicated that Fe–O and O–H on the surface of nZVI-RSBC might have participated in the adsorption of phosphate. The oxygen-containing functional groups on the surface of nZVI-RSBC (such as Fe–O and O–H) may act as hydrogen donors and acceptor groups and form strong hydrogen bonds with phosphate.

Typical thermogravimetric curves of RSBC and nZVI-RSBC are displayed in Fig. 2b. Three stages of mass loss were observed for RSBC and nZVI-RSBC: (1) <280 °C, nonstructural and free water loss; (2) 280–520 °C, thermal oxidation of organic carbon structures (such as graphitic and amorphous carbon); and (3) >520 °C, the weight of RSBC and nZVI-RSBC remained stable, and the residual mass could be attributed to the mineral content of RSBC and nZVI-RSBC.^29^ The residual mass of nZVI-RSBC is higher than that of RSBC. This is mainly because there are more iron oxides in nZVI-RSBC, which is consistent with the results in Table 2.

**Table:** Elemental composition, BET surface area, total pore volume and average pore size of RSBC and nZVI-RSBC

Sample	C (%)	H (%)	N (%)	Fe (%)	BET surface area (m^2^ g^−1^)	Total pore volume (cm^3^ g^−1^)	Average pore size (nm)
RSBC	64.48	3.72	0.74	0.07	1.21	0.0018	6.1
nZVI-RSBC	50.94	3.36	0.79	8.26	34.23	0.086	10.1

The N_2_ adsorption–desorption isotherms and pore size distribution of the RSBC and nZVI-RSBC are presented in Fig. 2c. The results showed that the pore volume and surface area of nZVI-RSBC were much higher than those of RSBC, and these results were consistent with the results in Table 2. The N_2_ adsorption isotherms of RSBC and nZVI-RSBC exhibited typical type-IV isotherms with distinct H3 type hysteresis loops at the relative pressure *p*/*p*_0_ of 0.45–0.95, demonstrating the presence of uniform mesopores in the samples.^27^ Compared with that of nZVI-RSBC, the hysteresis loop area of RSBC was smaller, which indicated that the incorporation of nZVI particles into the pores and crevices of RSBC or on the surface increased its porosity.

The BET surface area, total pore volume and average pore size of RSBC and nZVI-RSBC are displayed in Table 2. The increase of BET surface area from 1.21 m^2^ g^−1^ to 34.23 m^2^ g^−1^, the increase of total pore volume from 0.0018 cm^3^ g^−1^ to 0.086 cm^3^ g^−1^, and the increase of average pore size from 6.1 nm to 10.1 nm were observed for nZVI-RSBC compared with to RSBC. The increase of BET surface area, total pore volume and average pore size were possible because of the nZVI particles supported on the surface or int the pores of RSBC.^4^ When nZVI particles were supported on FSBC, the BET surface area of nZVI-RSBC was much higher than that of RSBC, suggesting that RSBC as a carrier played a critical role in the process of dispersing nZVI, thereby increasing the BET surface area and reactive sites.

**Fig. 3:**
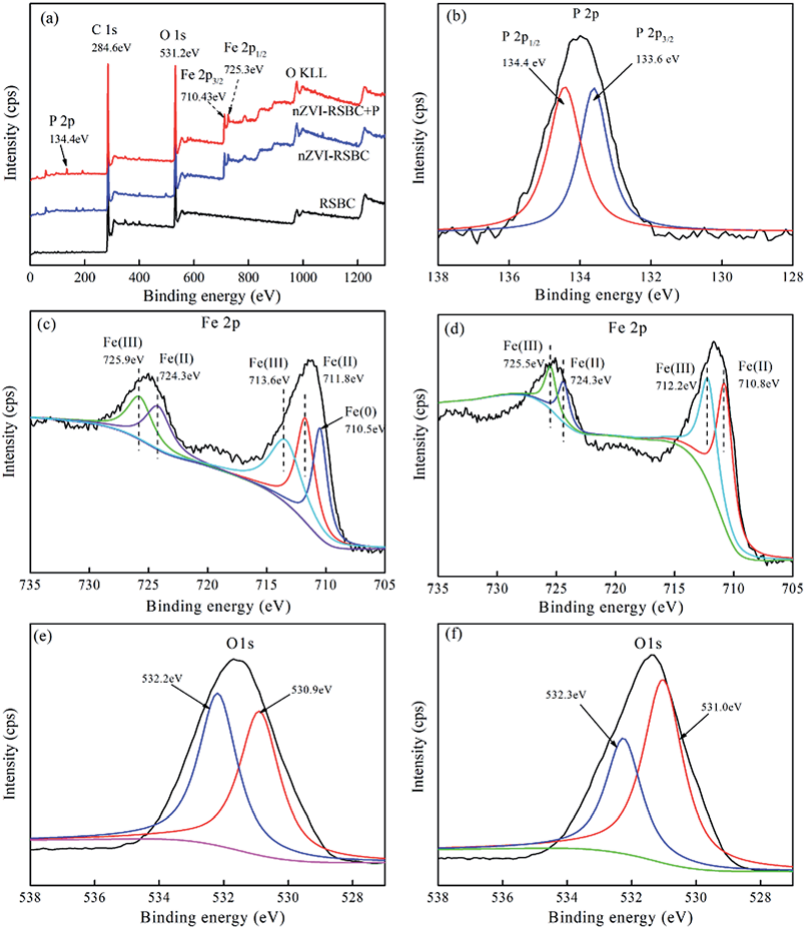
The XPS survey spectrum of RSBC and nZVI-RSBC, and nZVI-FSBC with phosphate adsorption (a); deconvolution of P 2p of nZVI-FSBC with phosphate adsorption (b); high-resolution XPS spectra of Fe 2p before (c) and after phosphate adsorption (d); high-resolution XPS spectra of O 1s before (e) and after phosphate adsorption (f).

To further investigate the mechanism of phosphate adsorption on nZVI-RSBC, XPS of nZVI-RSBC and phosphate loaded nZVI-RSBC are presented in Fig. 3. In the XPS survey spectra of nZVI-RSBC with phosphate adsorption (Fig. 3a), the peaks at 284.6 eV, 531.2 eV, 710.43 eV, and 134.4 eV were assigned to C, O, Fe, and P elements, respectively. Comparing the wide scan XPS of RSBC with that of nZVI-RSBC (Fig. 3a), it can be clearly found that new peaks appeared on the surface of nZVI-RSBC at 710.43 eV and 725.3 eV, which corresponded to Fe 2p_3/2_ and Fe 2p_1/2_, proving that nZVI particles were successfully supported on the surface of RSBC. The high resolution XPS spectrum of Fe 2p before and after phosphate adsorption are displayed in Fig. 3c and d. It can be seen that there are large portions of Fe(ii) and Fe(iii) on the surface of nZVI-RSBC, which may be caused by the partly oxidized shell over the Fe^0^ core, which was deeper (3–5 nm) than the XPS sampling depth.^30^ The O 1s spectrum of nZVI-RSBC before adsorption is divided into two peaks at 532.2 eV and 530.9 eV (Fig. 3e), indicating that different kinds of oxygen-containing functional groups (such as C=O and C–O) are presented on the nZVI-RSBC surface.^31^

### 3.2. Adsorption kinetics

**Fig. 4:**
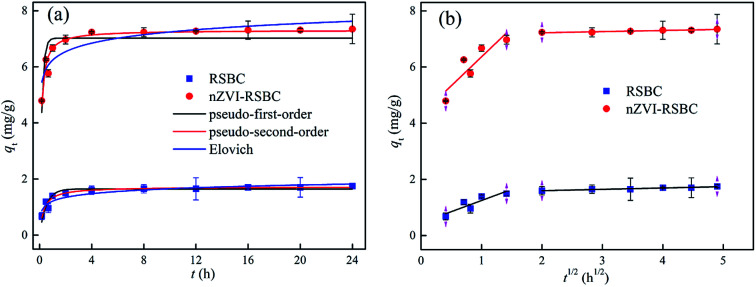
Fitting of Elovich, pseudo-first- and pseudo-second-order models for adsorption of phosphate on RSBC and nZVI-RSBC (a); intra-particle diffusion plots for adsorption of phosphate on RSBC and nZVI-RSBC (b) (pH 7.0, initial phosphate concentration 20 mg L^−1^, liquid volume 20 mL, RSBC and nZVI-RSBC dose 0.05 g).

The adsorption rate curves of phosphate on RSBC and nZVI-RSBC are shown in Fig. 4a. The results showed that the adsorption rates of phosphate on RSBC and nZVI-RSBC were very high and achieved apparent equilibrium in 3 h. It is expected that the available active sites on the adsorbent (RSBC and nZVI-RSBC) will gradually saturate over time, resulting in the slow adsorption of phosphate onto a great part of the adsorbent. The adsorption rate decreased over time because the empty surface sites of adsorbent were increasingly occupied by phosphate.

The kinetic data of phosphate on RSBC and nZVI-RSBC were fitted by four different models, and the results are presented in Table 3. From the regression coefficients (*R*^2^ values) of these models, the pseudo-second-order model was more appropriate than the other models to describe the phosphate adsorption kinetic behavior on RSBC and nZVI-RSBC. Besides, the value of *q*_m_ obtained by fitting the pseudo-second-order kinetic model was also shown to be very close to the value of *q*_e,exp_ observed in the experiment. This result suggested that chemical adsorption may be the rate control mechanism of phosphate adsorption on RSBC and nZVI-RSBC.^21^

Kinetic parameters for the adsorption of phosphate onto RSBC and nZVI-RSBC

**Table:** 

Adsorbents	Pseudo-first-order			Pseudo-second-order			Elovich		
	*q*_m_ (mg g^−1^)	*k*_1_ (h^−1^)	*R* ^2^	*q*_m_ (mg g^−1^)	*k*_2_ (g mg^−1^ h^−1^)	*R* ^2^	*α* (mg g^−1^ h^−1^)	*β* (g mg^−1^)	*R* ^2^
RSBC	1.65	2.01	0.849	1.72	1.97	0.929	1.21	5.23	0.848
nZVI-RSBC	7.02	5.86	0.658	7.31	1.41	0.928	6.24	2.29	0.785

**Table:** 

Intra-particle diffusion						
Adsorbents	*k*_d1_	*C*_1_	*R* ^2^	*k*_d2_	*C*_2_	*R* ^2^
RSBC	0.79	0.45	0.732	0.048	1.51	0.913
nZVI-RSBC	2.06	4.31	0.739	0.041	7.14	0.891

The intra-particle diffusion model was then used to analyze the adsorption rate-determining step of phosphate on RSBC and nZVI-RSBC. Fig. 4b shows that the intra-particle diffusion plots could be divided into two linear parts, indicating that the adsorption process of phosphate on RSBC and nZVI-RSBC may include two steps. The adsorption rate in the first step was higher (*k*_d1_ > 0.79, Table 3), and this step was limited by the diffusion of the external film because phosphate diffused through the boundary layer and adsorbed on the surface of the adsorbent (RSBC and nZVI-RSBC).^9^ The adsorption rate was significantly reduced (*k*_d2_ < 0.05, Table 3) in the second step, indicating that intra-particle diffusion was the primary rate-controlling step.^21^ In addition, the fitting curves of the two stages did not pass through the origin of the coordinate system, indicating that the adsorption process not only depended on intra-particle diffusion but was also determined by some surface complexation reactions.

### 3.3. Adsorption isotherms

**Fig. 5:**
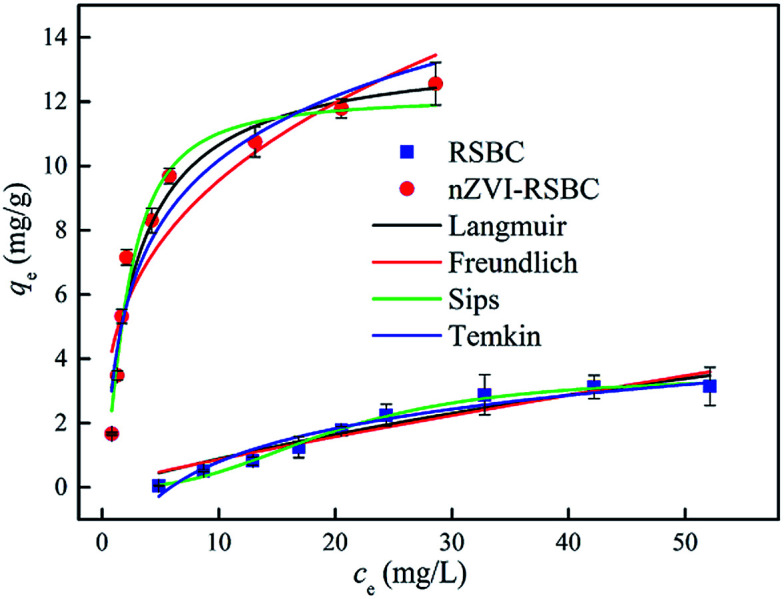
Adsorption isotherms of phosphate on RSBC and nZVI-RSBC.

The adsorption isotherms of RSBC and nZVI-RSBC were examined by changing the initial phosphate concentration in the range of 10–500 mg L^−1^ (Fig. 5). The data of adsorption isotherms were fitted by four different models, namely Langmuir, Freundlich, Sips, and Temkin model, and the results are shown in Table 4. According to the *R*^2^ values, the adsorption isotherm data could be better fitted by Sips and Langmuir models than the other models. The *q*_m_ values of RSBC and nZVI-RSBC evaluated by the Sips model (3.49 mg g^−1^ and 12.14 mg g^−1^, respectively) corresponded better to the experimentally obtained *q*_e,exp_ (3.14 mg g^−1^ and 12.56 mg g^−1^, respectively) than those evaluated by the Langmuir model (7.27 mg g^−1^ and 13.64 mg g^−1^). The Sips isotherm model is a combination of the Langmuir and Freundlich isotherm models, and is a three-parameter isotherm model. For the Sips model, if the value of *K*_S_ is close to zero, it follows the Freundlich isotherm model.^32^ In this study, the *K*_S_ value obtained by fitting the adsorption isotherm of RSBC adsorbed phosphate was 3.27 × 10^−4^, which was very small; thus, the Sips isotherm model followed the Freundlich model, suggesting that the adsorption of phosphate on RSBC was a heterogeneous multilayer adsorption process.^21^ However, the *K*_S_ value and *K*_L_ value fitted by the adsorption isotherm of nZVI-RSBC adsorbed phosphate were similar, indicating that the adsorption of phosphate on the nZVI-RSBC surface was a monolayer adsorption process. The parameter values obtained from the Temkin isotherm were: *A* < 2.9 L mg^−1^ and *K*_*t*_ < 3.5 J mol^−1^, demonstrating that the adsorption of phosphate onto RSBC and nZVI-RSBC occurred *via* physisorption. In the physisorption process, phosphate binds to the RSBC and nZVI-RSBC through a weak van der Waals interactions, so the process is related to relatively low adsorption energies.^33^ In addition, the adsorption capacity of nZVI-RSBC towards phosphate was significantly higher than that of RSBC. This may be because oxygen-containing functional groups on nZVI-RSBC are more abundant than on RSBC; these groups increase the number of active sites and thereby form iron oxide on the surface of nZVI-RSBC to form complexes with phosphate,^14^ thus increasing the affinity of the binding site and the energy of nZVI-RSBC. The maximum adsorption capacity of nZVI-RSBC in this study were higher than some other biochar adsorbents and adsorption materials in literatures (0.92–12.1 mg g^−1^),^34–38^ with the exception of Mg-biochar from ground coffee waste (56 mg g^−1^),^39^ sewage sludge-based phosphate adsorbent (93.91 mg g^−1^),^40^ Ce^3+^-enriched adsorbent (77.7 mg g^−1^),^41^ and Al-modified biochar (57.49 mg g^−1^).^42^

**Table:** Isotherm parameters of phosphate adsorption on RSBC and nZVI-RSBC

	Langmuir			Freundlich			Sips				Temkin		
Adsorbents	*q*_m_ (mg g^−1^)	*K*_L_ (L mg^−1^)	*R* ^2^	*K*_F_ (mg g^−1^)	*n*	*R* ^2^	*q*_m_ (mg g^−1^)	*K*_S_ (L mg^−1^)	*n*	*R* ^2^	*A* (L mg^−1^)	*K*_*t*_ (J mol^−1^)	*R* ^2^
RSBC	7.27	0.008	0.922	0.12	1.16	0.903	3.49	3.27 × 10^−4^	0.37	0.991	1.49	0.17	0.946
nZVI-RSBC	13.64	0.36	0.949	4.51	3.06	0.843	12.14	0.32	0.68	0.961	2.88	3.44	0.931

### 3.4 Adsorption mechanism analysis

#### 3.4.1 Effect of pH value of solution

**Fig. 6:**
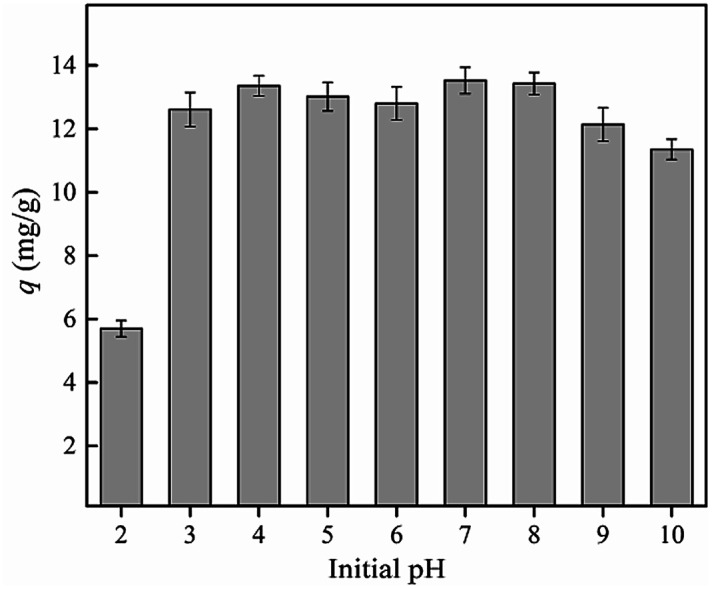
Effect of initial pH value of solution on phosphate adsorption by nZVI-RSBC.

The pH value of solution is one of the key environmental factors affecting the behavior of phosphate adsorption, which affects not only the form of surface functional groups on the surface of the adsorbent, but also the conditions of the phosphate ions in the aqueous solution.^11^ The effect of the pH value of solution on the adsorption of phosphate to nZVI-RSBC is presented in Fig. 6. The adsorption capacities of nZVI-RSBC remained almost unvaried in the pH range of 3–8 but decreased at a pH of 2 and greater than 8, indicating that nZVI-RSBC has a strong anti-pH-interference ability.^43^ The wide pH tolerance range of nZVI-RSBC greatly expands its application range. The adsorption of phosphate on nZVI-RSBC reached the maximum adsorption capacity when the pH value of solution was in the range of 3 to 8, mainly because in addition to hydrogen bonding ability and electrostatic attraction, the addition of nZVI also increased the adsorption of phosphate by enhancing the complexation.

The main reason why the adsorption capacity of nZVI-RSBC towards phosphate changes with the variation of the pH value of the solution was determined by the surface charge of nZVI-RSBC and the distribution of phosphate species. Phosphate is polyacidic (p*K*_1_ = 2.12, p*K*_2_ = 7.21, p*K*_3_ = 12.67) and often exists as different species, which mainly depends on the pH value of solution (*i.e.*, HPO_4_^2−^ when 7.21 ≤ pH ≤ 12.67 and H_2_PO_4_^−^ when 2.12 ≤ pH ≤ 7.21).^44^ The isoelectric point (IEP) of nZVI-RSBC was 7.1. When the pH of the solution is lower than 7.1, the surface of nZVI-RSBC is positively charged, which facilitates the electrostatic attraction between nZVI-RSBC and H_2_PO_4_.^44^ As the pH value of solution further exceeds 7.1, the surface of nZVI-RSBC became negatively charged, which was not conducive to the electrostatic attraction of nZVI-RSBC and HPO_4_^2−^. It can be seen from Fig. 6 that when the pH value of the solution was greater than 9, stronger electrostatic repulsion and OH^−^ competitive adsorption may contribute to this reduction of phosphate adsorption capacity on nZVI-RSBC.^45^ These results revealed that the electrostatic attraction of phosphate on nZVI-RSBC was controlled by the interactions of negatively or positively surface charged nZVI-RSBC with the divalent or monovalent anionic phosphate species. The strength and direction of the interaction between the surface of nZVI-RSBC and the phosphate species were significantly changed with the pH value of the solution.^1^

#### 3.4.2 XPS spectra

To further understand the mechanism of the adsorption of phosphate by nZVI-RSBC, XPS surveys before and after phosphate adsorption were conducted and the results are shown in Fig. 3. From the wide scan XPS spectrum (Fig. 3a), a new obvious peak at 134.4 eV ascribed to P 2p was observed on the surface of nZVI-RSBC after phosphate adsorption, indicating that phosphate was successfully adsorbed on the surface of nZVI-RSBC.^46^ As shown in Fig. 3b, the P 2p XPS spectrum of nZVI-RSBC after phosphate adsorption was deconvoluted into two peaks ascribed to O–P interaction (134.4 eV) and Fe–P interaction (133.6 eV), respectively, suggesting the formation of complexes between phosphate and nZVI-RSBC.^47^ The detailed spectra of Fe 2p before and after phosphate adsorption are depicted in Fig. 3c and d, respectively. Iron species with different oxidation states, namely Fe(0), Fe(ii) and Fe(iii), are synchronously presented. The peaks at 724.3 eV and 711.8 eV were associated with the Fe(ii) species, whereas the peaks at 725.9 eV and 713.6 eV were ascribed to the Fe(iii) species.^31^ The peak at 710.5 eV was assigned to the Fe(0) species.^48^ The presence of Fe(0) and thin oxide layer before phosphate adsorption confirmed the core–shell structure on the surface of nZVI-RSBC. After phosphate adsorption, the peak at 710.5 eV corresponding to Fe(0) disappeared, demonstrating that Fe(0) on nZVI-RSBC was completely oxidized.^49^ The calculated ratio of Fe(iii)/Fe(ii) of nZVI-RSBC increased significantly after phosphate adsorption, indicating that the amount of Fe(ii) decreased while the ratio of Fe(iii) increased.

As shown in Fig. 3e and f, the O 1s spectra before and after phosphate adsorption could be deconvoluted into two peaks at 532.2 eV and 530.9 eV, corresponding to Fe–OH (hydroxyl group) and Fe–O (oxide oxygen), respectively.^50^ According to the fitting data recorded by XPSPEAK4.1 software, the peak corresponding to the Fe–OH species at 532.2 eV decreased from 50.2% to 36.1%, while the peak corresponding to Fe–O at 530.9 eV increased from 49.8% to 63.9% after phosphate adsorption. This result further indicated that the oxygen-containing functional groups on the surface of nZVI-RSBC played a significant role in the process of phosphate adsorption,^1^ in agreement with the FTIR results. The XPS and FTIR results revealed that complexation was the dominant mechanism for phosphate adsorption.

Based on the effect of the pH value of solution on the adsorption mechanism and the FTIR and XPS characterization results of nZVI-RSBC after phosphate adsorption, the adsorption mechanism of phosphate on nZVI-RSBC was hydrogen bonding between the oxygen-containing functional groups on the surface of nZVI-RSBC and phosphate. The observation that the combination of RSBC and nZVI can enhance the adsorption capacity of phosphate was caused by complexation and electrostatic interactions. It could be concluded that the synergy of RSBC and nZVI was considerable for phosphate removal. In general, the mechanism of nZVI-RSBC to remove phosphate from aqueous solution seems to mainly involve complexation, hydrogen bonding, and electrostatic interaction.

### 3.5. Reusability test of nZVI-RSBC

**Fig. 7:**
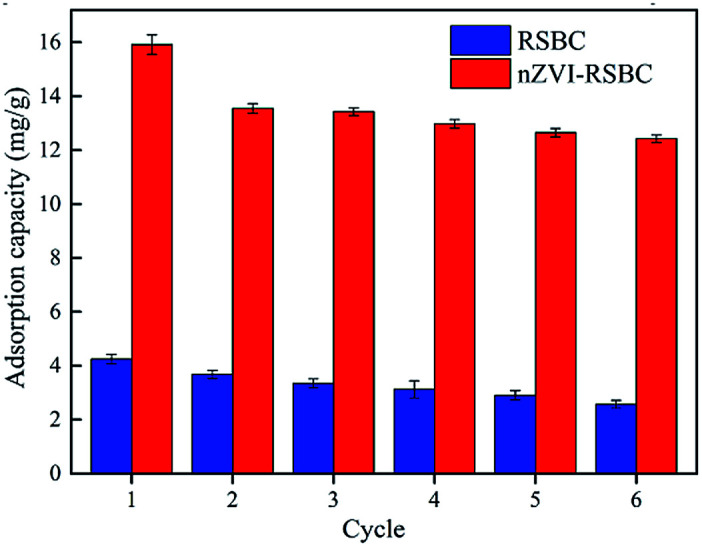
Adsorption and desorption cycles performance of RSBC and nZVI-RSBC for phosphate uptake.

It is important that effective desorption must be carried out after absorbing phosphate to realize the economic value of nZVI-RSBC. In this study, RSBC and nZVI-RSBC were subjected to 6 cycles of phosphate adsorption–desorption using 1.0 M NaOH as the eluent. The adsorption capacity after each cycle was measured to assess the reusability of RSBC and nZVI-RSBC, and the results are illustrated in Fig. 7. Obviously, the adsorption capacity of RSBC and nZVI-RSBC for phosphate gradually decreased as the number of repeated runs increased, which was due to the active sites being permanently filled after the subsequent adsorption cycle. After six consecutive adsorption–desorption sequences, the adsorption capacity of RSBC and nZVI-RSBC for phosphate dropped significantly by 40% and 22%, respectively. Therefore, nZVI-RSBC could be regarded as a reliable adsorbent for phosphate removal from an economic perspective.

## 4 Conclusions

This study investigated the potential of nZVI-RSBC as an adsorbent to remove phosphate from aqueous solutions. The results indicated that nZVI-RSBC had a high specific surface area and abundant oxygen-containing functional groups. The adsorption kinetic data were well fitted by the pseudo-second-order kinetic model, and the intra-particle diffusion model showed that the adsorption process was controlled by intra-particle diffusion and film diffusion. The adsorption capacity of nZVI-RSBC for phosphate was significantly higher than the adsorption capacity of RSBC for phosphate, *i.e.*, 3.5 times that of RSBC. The adsorption mechanism of nZVI-RSBC towards phosphate included complexation, hydrogen bonding, and electrostatic interaction. The result of this mechanism provides a basis for future modification of the adsorbent material that is more capable of adsorbing phosphate. Moreover, from an economic point of view, crude biochar prepared by pyrolyzing waste agricultural rape straw is easy to obtain and inexpensive, and could effectively solve the problem of the disposal of waste agricultural rape straw. Therefore, nZVI-RSBC as a potential adsorbent plays an important role in removing phosphate from aqueous solution.

## Conflicts of interest

There are no conflicts to declare.

## Supplementary Material

RA-010-D0RA07391A-s001
