# Supplementary material for: Mechanism of phosphate removal from aqueous solutions by biochar supported nanoscale zero-valent iron
Source: RSC Adv. 2020 Oct 26;10(64):39217–25. doi: 10.1039/d0ra07391a (PMC9057329; doi:10.1039/d0ra07391a)
Supplement: RA-010-D0RA07391A-s001 [file RA-010-D0RA07391A-s001.pdf]

Electronic Supplementary Material (ESI) for RSC Advances.
This journal is © The Royal Society of Chemistry 2020

Electronic supplementary information

Mechanism of phosphate removal from aqueous solutions by biochar supported nanoscale zero-valent iron

Fengfeng Ma*, Baowei Zhao, Jingru Diao, Yufeng Jiang, Jian Zhang

(School of Environmental and Municipal Engineering, Lanzhou Jiaotong University, Lanzhou 730070, China)

*Corresponding author. E-mail: mayibo1985@126.com

TEXT S1

Adsorption models

Table 1. List of adsorption kinetic and isotherm models used in this study

Models	Expression
pseudo-first-order	$\frac{dq_t}{dt} = k_1(q_e - q_t)$
pseudo-second-order	$\frac{dq_t}{dt} = k_2(q_e - q_t)^2$
Elovich	$\frac{dq_t}{dt} = \alpha \exp(-\beta q_t)$
Intra-particle diffusion	$q_t = k_d t^{1/2} + C_i$
Langmuir	$q_e = \frac{q_m K_L c_e}{1 + K_L c_e}$
Freundlich	$q_e = K_F c_e^{1/n}$
Sips	$q_e = \frac{q_m K_S c_e^{1/n}}{1 + K_S c_e^{1/n}}$
Temkin	$q_e = A \ln K_t c_e$

where q_t and q_e (mg/g) are the amounts of phosphate adsorbed at time t (h) and equilibrium, respectively; k_1(h^{-1}), k_2 (g/mg/h), and k_d (mg/g/h$^{-1/2}$) are the pseudo-first-order, pseudo-second-order, and intra-particle diffusion rate constants, respectively; α (mg/g/h) is the initial adsorption rate, β (g/mg) is the desorption constant, and C_i is a constant related to the boundary layer thickness; K_L (L/mg), K_F (L/mg), K_S, and K_t are the Langmuir, Freundlich, Sips, and Temkin constants, respectively.

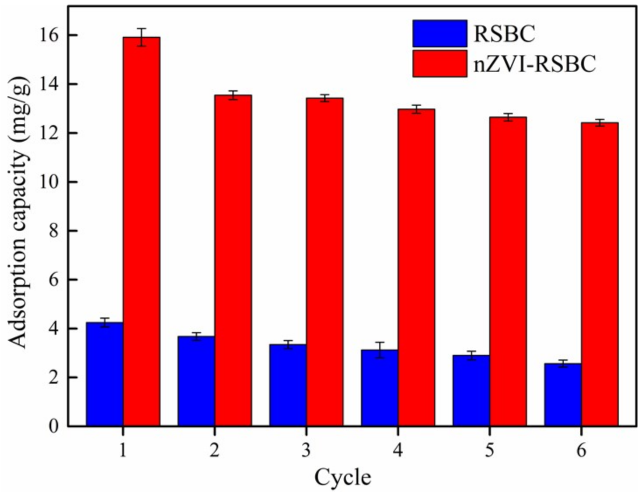

Fig. S1 Adsorption and desorption cycles performance of RSBC and nZVI-RSBC for phosphate uptake
